# Two-dimensional gel proteome reference map of blood monocytes

**DOI:** 10.1186/1477-5956-4-16

**Published:** 2006-09-01

**Authors:** Ming Jin, Philip T Diaz, Tran Bourgeois, Charis Eng, Clay B Marsh, Haifeng M Wu

**Affiliations:** 1 Department of Pathology, The Ohio State University, Columbus, OH, USA; 2 Division of Pulmonary, Critical Care and Sleep Medicine, Department of Internal Medicine, The Ohio State University, Columbus, OH, USA; 3 Dorothy M. Davis Heart and Lung Research Institute, The Ohio State University, Columbus, OH, USA; 4 Genomic Medicine Institute, Lerner Research Institute, Cleveland Clinic Foundation, Cleveland, OH, USA; 5 Department of Genetics, Case Western Reserve University School of Medicine, Cleveland Clinic Foundation, Cleveland, OH, USA

## Abstract

**Background:**

Blood monocytes play a central role in regulating host inflammatory processes through chemotaxis, phagocytosis, and cytokine production. However, the molecular details underlying these diverse functions are not completely understood. Understanding the proteomes of blood monocytes will provide new insights into their biological role in health and diseases.

**Results:**

In this study, monocytes were isolated from five healthy donors. Whole monocyte lysates from each donor were then analyzed by 2D gel electrophoresis, and proteins were detected using Sypro Ruby fluorescence and then examined for phosphoproteomes using ProQ phospho-protein fluorescence dye. Between 1525 and 1769 protein spots on each 2D gel were matched, analyzed, and quantified. Abundant protein spots were then subjected to analysis by mass spectrometry. This report describes the protein identities of 231 monocyte protein spots, which represent 164 distinct proteins and their respective isoforms or subunits. Some of these proteins had not been previously characterized at the protein level in monocytes. Among the 231 protein spots, 19 proteins revealed distinct modification by protein phosphorylation.

**Conclusion:**

The results of this study offer the most detailed monocyte proteomic database to date and provide new perspectives into the study of monocyte biology.

## Background

Blood monocytes belong to the human mononuclear phagocyte system which plays an important role in a variety of homeostatic processes including host defense, immunoregulation, and tumor surveillance [1-3]. Derived from bone marrow monoblasts, monocytes circulate in the blood for 1–2 days and then enter various tissues to differentiate into macrophages that exhibit specific activities [4-6]. Blood monocytes regulate host inflammatory processes through chemotaxis, pinocytosis/phagocytosis, and the release of cytokines [1,2,7,8]. However, the molecular details underlying these diverse biological activities are not well defined. Recent advances in proteomics have made it possible to gain a global perspective of the protein constituents and their properties in blood monocytes. Analysis of a monocyte proteome and phosphoproteome enables us to learn more about monocyte biology and better understand the mechanisms of diseases involving blood monocytes.

One previous study demonstrated the feasibility of monocyte proteome analysis by 2D electrophoresis but did not provide protein ID information [9]. Another study revealed 16 monocyte proteins that demonstrated significant level changes after stimulating monocytes with lipopolysaccharide [10]. In this study, more than 1412 monocyte protein spots among five healthy donors were matched, quantified, and analyzed. Through this process, 231 protein spots, representing 164 proteins and their respective isoforms or subunits, were identified using tandem mass spectrometry. Furthermore, phosphorylation states of these proteins were determined. This study provides, for the first time, a detailed profile of blood monocyte proteomes and phosphorylation proteomes critical for future studies of monocyte biology and pathophysiology. The biochemical characteristics of many proteins are revealed for the first time in blood monocytes, providing a new roadmap for functional investigations of monocyte biology.

## Results

Fresh buffy coats were obtained from five healthy donors between the ages of 18 and 55 with a gender ratio of 3:2 (M:F). Since the aim of this initial study is to establish monocyte proteome reference among the general population, information on ethnicity was not recorded. However, the potential influence of ethnicity on proteome variability should be considered when monocyte proteomes are analyzed during epidemiological and translational investigations in the future.

**Figure 1:**
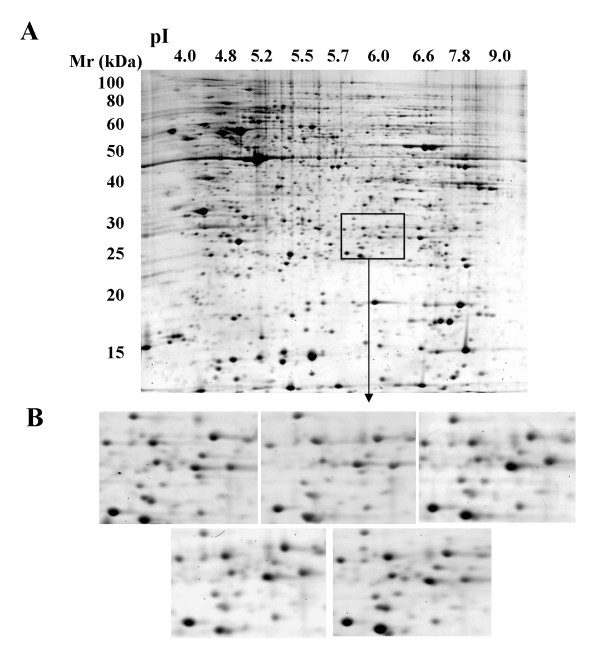
**Demonstration of monocyte 2D gel reproducibility between runs and visual examination of proteome analysis of all five 2D gels using the montage window of a specific gel area in blood monocytes**. (A) Monocyte 2D gel from donor 1. (B) Magnified image of the same gel area for all five monocyte 2D gels.

Resultant protein spots obtained from all five 2D gels ranged from 1525 – 1769. All gels were matched to the monocyte reference 2D map and the relative density of each protein spot quantified using ImageMaster software. Figure 1 depicts the same area for five 2D gels generated respectively from the monocytes of five donors, emphasizing the high level of protein resolution and reproducibility achieved in this experimental process. The excellent reproduction in protein pattern and protein density between all donors is evident in the comparison of five 2D gels to each other.

**Figure 2:**
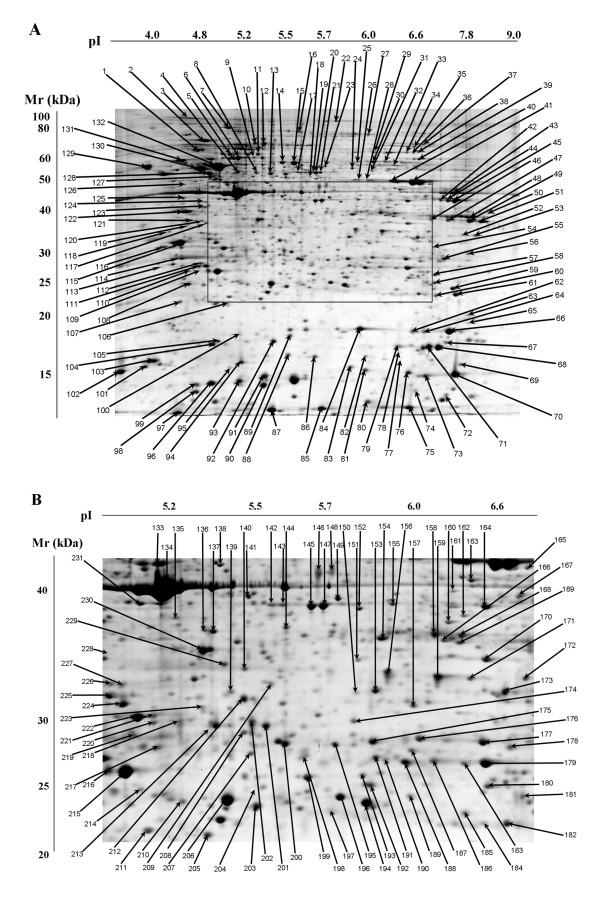
**Monocyte proteome map**. (A) 2D gel of blood monocytes. Arrows point to abundant proteins with protein identity determined by tandem mass spectrometry. (B) Detailed illustration of the central area of Figure 2A. Arrows illustrate protein spots in the central area that have information on protein identity.

The focus of this study was to identify the most abundant monocyte protein spots and define their biochemical characteristics. To ensure accuracy of the protein ID process, each spot was subjected to MALDI TOF/TOF analysis twice for confirmation of each protein identity. Two hundred thirty-one protein spots successfully identified and reconfirmed with high confidence (MOWSE score >50) are shown in Table 1 [see Additional file 1]. Other protein spots either failed identity reconfirmation or exhibited low confidence scores on repeated attempts (MOWSE score <50). This initial analysis did not include low abundance protein spots because of anticipated difficulties in obtaining satisfactory protein identification by mass spectrometry. For practical purposes, we arbitrarily define any spots with a density <0.02% of total protein density in the gel as "low abundance proteins". Figure 2 shows the reference monocyte 2D gel chosen from this experiment. The arrow and spot number indicate successfully identified spots present in monocytes. Protein spot numbers in Table 1 [see Additional file 1] correspond to the spots shown in Figure 2. Also listed in Table 1 [see Additional file 1] are the biochemical features of these proteins including pI, M_r_, relative protein amount (mean ± SD), phosphorylation status and variability of protein densities among five donors.

A PUBMED search revealed some proteins in Table 1 [see Additional file 1] that have yet to be characterized in blood monocytes. Each candidate protein was examined using Swiss-Prot/TrEMBL database with respect to their known tissue specificity. After excluding the proteins with known ubiquitous tissue distribution, eight proteins were identified as monocyte proteins that had not been characterized in blood monocytes at the protein level. These interesting proteins include swiprosin 1, Thyroid receptor interacting protein 7, and several putative proteins such as Novel Protein c20orf178, which provide new perspectives in monocyte biology.

**Figure 3:**
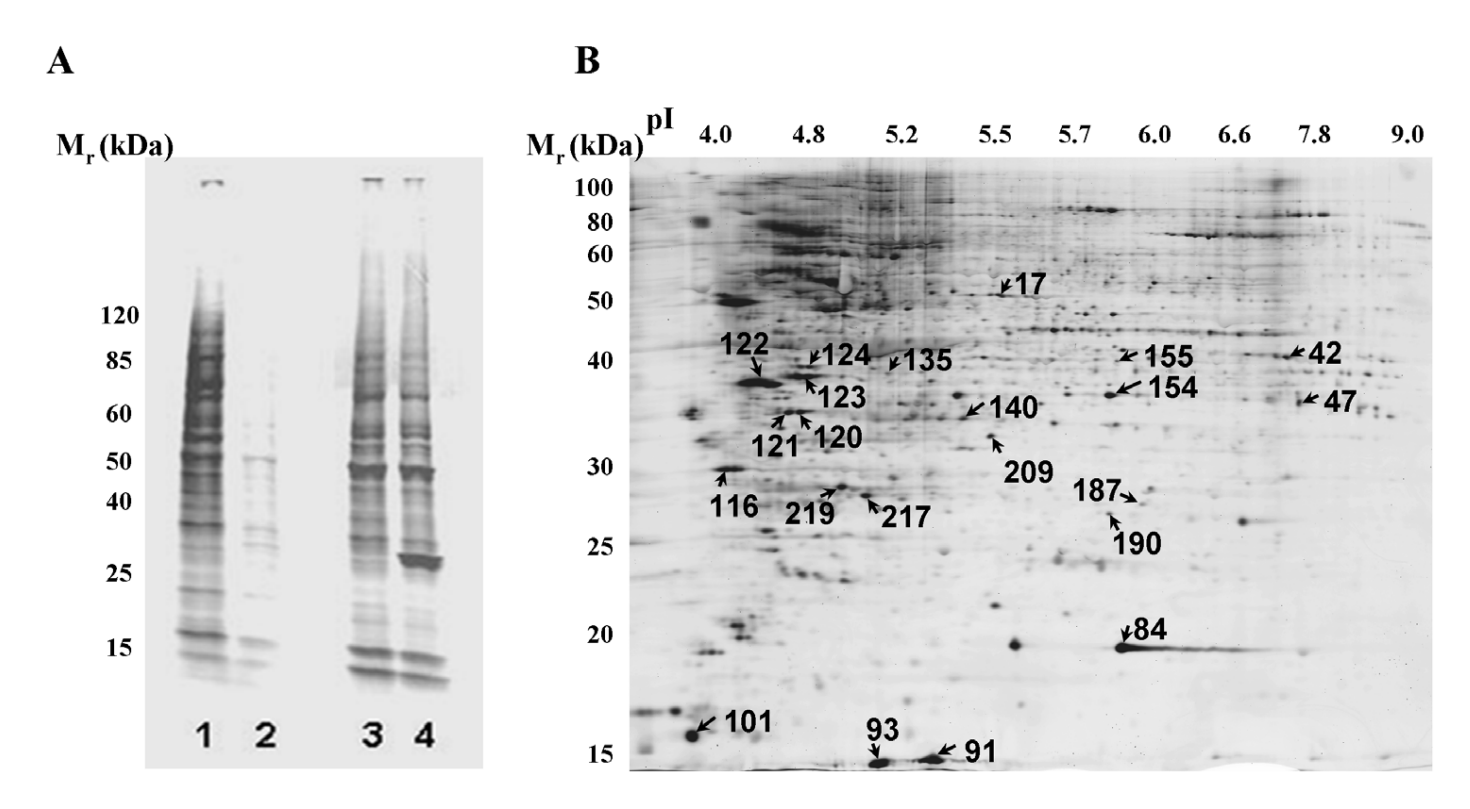
**Analysis of monocyte phosphoproteome by ProQ staining**. (A) Specificity of pro Q stain for phosphorylated proteins. Lane 1: Pro Q staining of monocyte lysates. Lane 2: Same cell lysates treated with protein phosphatase (80 units/ml) for 60 minutes at 30°C prior to SDS-PAGE/Pro Q staining. Lane 3: Sypro Ruby fluorescence staining of Lane 1. Lane 4: Sypro Ruby fluorescence staining of Lane 2. A protein band at about 25 kDa is consistent with M_r _of protein phosphatase added to the sample. (B) A 2D gel of monocytes stained with Pro Q diamond phosphoprotein dye. Arrows point to the protein spots with IDs and positive for phosphorylation stain.

All five 2D gels were also subjected to staining by Pro-Q phospho-protein fluorescence dye in order to generate more biological insight into the monocyte proteome through evaluation of the phosphorylation statuses of all protein spots identified. These phosphor-images were matched to the monocyte reference 2D gel in this experiment, thus, phosphorylation status of all these protein spots (Table 1) [see Additional file 1] was evaluated. Nineteen proteins that demonstrated distinct modification by phosphorylation in all five 2D gels are described in Table 1 [see Additional file 1] and in Figure 3.

## Discussion

Blood monocytes are key inflammatory cells. Global analysis of the monocyte proteomes will offer new perspectives into understanding host defense and inflammatory responses. A reference 2D gel map of blood monocytes was developed to promote future studies of blood monocyte proteomics. This map contains protein identities of 231 spots, representing 164 proteins and their respective isoforms or subunits and their biochemical properties. Through this analysis, many protein's biochemical properties such as pI value, M_r_, and relative quantity are revealed for the first time in blood monocytes. Importantly, several proteins, including novel proteins predicted by the Human Genome Project, were first characterized in blood monocytes, providing a new roadmap for studies on monocyte biology. Furthermore, high protein resolution of 2D gel electrophoresis showed that many monocyte proteins exhibited multiple isoforms, creating a basis for studying the biological impact attributed by different protein isoforms.

Post-translational protein modification by phosphorylation plays a central role in transducing signals responsible for a variety of biological processes. In monocytes, the expression of cellular activities involves numerous protein kinases and their downstream-phosphorylated substrates [11-13]. Pro Q dye allows for fluorescent detection of phosphorylated amino acids with a detection sensitivity of 0.1 f*mole *of mono-phosphorylated proteins [14-17]. This procedure thus offers a feasible approach to detecting general protein phosphorylation states either at Tyr-, Ser-, or Thr-residue(s). It was previously predicted that one-third of all proteins in eukaryotic cells are phosphorylated at any given time [14,15]. For each monocyte sample separated by 2D gel, 400 to 600 phosphorylated protein spots were found; which is about one-third of the number of proteins detected on a Sypro Ruby gel. However, our final results showed that only 22 out of 231 protein spots, representing nineteen distinct proteins, were phosphorylated in all five Pro Q stained 2D gels. A detailed data analysis showed that this smaller percentage of proteins identified as phosphorylated in Table 1 [see Additional file 1] was due to the following factors. First, the detection sensitivity of Pro Q fluorescence dye exceeded that of Sypro Ruby staining because many phosphorylated spots on Pro Q stained 2D gels did not have corresponding spots on the 2D gel stained with Sypro Ruby dye. Thus, many phosphorylated proteins were of low abundance, making protein identity determination difficult. Second, some proteins showed protein modification by phosphorylation in only a fraction of donors. These proteins were not included in the final list of phosphorylated proteins, because it is unclear whether these differences in protein phosphorylation between individuals contributed to biological variation or arose from *in vitro *artifact. Further studies are required to clarify the biological implication of these inter-individual phosphorylation differences. Third, many phosphorylated proteins tend to cluster together in the acidic pH region, making it difficult to perform protein quantification and analysis. These poorly separated phosphorylated proteins were not included in Table 1 [see Additional file 1]. Future studies using narrower pH ranges during first D electrophoresis will be necessary to improve resolution of these phosphorylated proteins. As a side note, the extent of protein phosphorylation, site of phosphorylation, and phosphorylation of specific amino acids are all-important in expressing protein function and activity. These topics should be addressed using mass spectrometry or a combination of 2D gel electrophoresis with Western blot analysis in future studies [18-20].

## Conclusion

In summary, blood monocytes are key inflammatory cells. Comprehension of the monocyte proteome will provide considerable new insight into the biology of monocytes and their roles in disease process. This study reported a comprehensive 2D gel proteome map of blood monocytes that contains information on the protein identities of 231 protein spots, their biochemical characteristics, and each protein's phosphorylation status. As a result, many proteins were characterized for the first time in blood monocytes, providing new clues to the study of monocyte biology. These rich datasets are therefore of considerable value for future studies in: 1) understanding the molecular basis of monocyte functions; 2) defining proteome features of blood monocytes in disease processes; and 3) phosphoproteomics of blood monocytes.

## Methods

### Materials

Ficoll gradient (Histopaque) was obtained from Sigma (St. Louis, MO). All other reagents for cell preparations, including RPMI-1640 (containing L-glutamine), FBS, and penicillin/streptomycin were obtained from Gibco (Grand Island, NY). For 2D gel experiments, SyproRuby Fluorescence dye and Pro Q phosphor-protein fluorescence dye came from Molecular Probes (Eugene, OR). Immobilized pH Gradient (IPG) buffer and IPG strips (pH 3–10) came from Amersham (Piscataway, NJ). Phosphatase and protease inhibitor cocktails were obtained from Boehringer Mannheim (Mannheim, Germany). DTT came from Invitrogen (Carlsbad, CA). Trypsin was obtained from Promega (Madison, WI). Lambda Protein Phosphatase was from New England BioLabs Inc. All other reagents and chemicals were obtained from Sigma unless otherwise noted.

### Donor characteristics

Blood monocytes were purified from fresh buffy coats obtained from five donors. Buffy coats were supplied by the American Red Cross in Columbus, OH under a research protocol approved by the IRB committee at both the Ohio State University and the American Red Cross. All buffy coats used in this study were freshly obtained and processed within 12 hours of blood collection. In addition, care was taken to not refrigerate buffy coats or monocytes during storage and sample processing because cold exposure may cause monocyte clumping. The five donors ranged in ages between 18 and 55 and exhibited a gender ratio of 3:2 (M:F). Racial information was not provided since the Red Cross IRB protocol prohibits disclosure of information pertaining to donor ethnicity.

### Isolation of peripheral blood monocytes

Isolation of blood monocytes was performed within 12 hours after blood was drawn from healthy donors. The detailed procedure was described previously [21]. A volume of approximately 60 ml buffy coat, derived from one unit of blood, was first diluted with an equal volume of PBS and then isolated by density gradient centrifugation over Histopaque. The mononuclear layer was pooled and resuspended in RPMI-1640. Monocytes were then isolated by negative selection using a monocyte isolation kit from Miltenyi Biotech (Auburn, CA). Monocyte purity in each cell preparation was evaluated by light microscopic examination of diff-quick cytospins to ensure at least 90% purity similar to that reported by others [9]. Contaminating cells consisted mainly of lymphocytes, with the percentage varying from 1 – 6% in all donors used in these studies. Monocyte numbers obtained from each donation ranged from 60 × 10^6 ^to 100 × 10^6 ^cells. In order to minimize possible monocyte activation due to in vitro sample manipulation, all procedures involving cell isolation were performed under sterile conditions and completed in a timely fashion. After isolation, the cells were immediately lysed in a cell lysis buffer containing both protease and phosphatase inhibitors. The sample was then frozen at -80°C until further analysis using electrophoresis.

### 2D gel electrophoresis

Isolated monocytes were washed with cold HBSS buffer. Afterwards, cell lyses were performed at a density of 25 × 10^6 ^cells per 100 μL cell lysis buffer. Cell lysates were set on a rotator at room temperature for 60 minutes and then spun at 14,000 rpm at 4°C for 20 minutes to remove cell debris and DNA aggregates. The supernatant was collected and frozen at -80°C for later 2D electrophoresis. For first D electrophoresis, 100 μl of the cell lysatescollected above were mixed with 350 μL rehydration buffer, and then loaded onto each IPG strip in 2D gel system. As a result, all 2D gels contained the amount of proteins solubilized from approximately 25 × 10^6 ^cells under our experimental conditions. Following first D electrophoresis, each strip was equilibrated and subjected to second D electrophoresis (12% SDS-PAGE) on an Amersham Dalt II system as described previously [21,22].

### Protein and phosphoprotein detection, quantification, and analysis

All five 2D gels were subjected to fluorescence staining with Pro-Q Diamond phosphoprotein dye (Molecular Probes) initially and then stained with SyproRuby for detection of proteins. Following 2D electrophoresis, Pro-Q stain was performed by first fixing the gels in 50% methanol/10% Trichloroacetic Acid (TCA) overnight. The gels were then washed with distilled water and subjected to Pro-Q Diamond phosphoprotein stain for 4 hours. Destaining was conducted with successive washes of 50 mM sodium acetate, pH 4.0 containing 15% 1,2-propanediol. Finally, a gel image was obtained by scanning the 2D gel on a Typhoon 9200 laser scanner (Amersham). The gels were then stained with SyproRuby fluorescence dye for protein detection as described previously [21]. Gel images were captured on a Typhoon 9200 laser scanner. Protein quantification on all five 2D gels was performed using ImageMaster 2D software (Non-linear Dynamics). At the time of setting-up image analysis file in the ImageMaster software, we selected a gel with good protein resolution, excellent protein stain, and minimal image distortion to be the reference gel. Afterwards, other 2D gels and the gels from later experiments were matched to the reference 2D gel for spot matching and analyses. Between 1525 and 1769 protein spots on each 2D gel were matched, analyzed, and quantified. On average, greater than 80% of all protein spots on each gel were successfully matched to their respective protein spot on the reference gel. For each protein spot in the reference gel, a molecular weight (M_r_) was assigned using M_r _standard markers (Invitrogen) and pI values were estimated based on manufacturer's instructions (Amersham). The density of each protein spot on a 2D gel was calculated as a percentage of total protein volume of the 2D gel in order to minimize variations caused by staining and destaining procedures between all 2D gels. Abundant spots were then cut by a Bio-Rad robotic 2D spotcutter platform and subjected to Mass Spectrometry (MS) analysis. Phosphoprotein images from Pro Q staining were matched to the reference monocyte 2D gel. Detection, quantification, and analysis of phosphorylated proteins were performed using ImageMaster 2D software.

### Protein identification by tandem Mass Spectrometry

Protein identification was performed using MALDI TOF/TOF (Applied Biosystems, Foster City, CA) as described previously [21,23]. The spots cut from each 2D gel were washed 2 times with water and then washed 3 times with 25 mM ammonium bicarbonate followed by acetonitrile. Once dehydrated with acetonitrile, the gels were then subjected to Trypsin digestion and concentrated using Millipore ZipTips. Any bound peptides were eluted onto a MALDI plate set in an 8 mg/ml solution of α-cyano-4-hydroxycinnamic acid in 50% acetonitrile/0.1% trifluoroacetic acid. Samples were then placed in the 4700 Proteomics Analyzer. MS and MS/MS spectra were obtained by 4700 Explorer™ Software v2.0. Trypsin was used as an internal standard, with prominent trypsin and keratin peaks excluded from subsequent MS/MS analysis. GPS Explorer v2.0 and Matrix Science Mascot v1.9 were used for database searching. Parameters used for searching were as follows: one missed cleavage, oxidation on methionine, and 0.3 Dalton mass tolerance. The resulting MS and MS/MS peptides were matched to the SwissProt, TrEMBL [25] and NCBI protein databases [26]. The threshold for positive identification was a MOWSE [24] score of >50. Each candidate ID derived from the above search was then manually examined in the SwissProt database to eliminate redundancy of synonymous proteins. A protein's name and accession number were reported based on SwissProt except for proteins that are only deposited in the NCBI database.

## Abbreviations

Immobilized pH Gradient: IPG; Molecular Weight: M_r_; Trichloroacetic Acid: TCA; Matrix Assisted Laser Desorption/Ionization Time of Flight/Time of Flight: MALDI TOF/TOF; Phosphate Buffered Saline: PBS; Hanks Balanced Salt Solution: HBSS.

## Competing interests

The author(s) declare that they have no competing interests.

## Authors' contributions

All authors read and approved the final manuscript.

MJ carried out the proteomic studies, data analysis, and participated in designing the study and drafting the manuscript.

PD participated in the design of the study, and helped to draft the manuscript.

TB participated in experiment, data analysis and helped to draft the manuscript.

CE participated in the design of the study, and helped to draft the manuscript.

CM participated in the design of the study, and helped to draft the manuscript.

HW designed the study, supervised experiments and data analysis, and drafted the manuscript.

## Supplementary Material

Additional file 1 Table 1. Abundant monocyte proteins, their biochemical characteristics, and phosphorylation states. Click here for file
